# Cultural Entertainment Consumption and Empathy Communication Mechanism

**DOI:** 10.3389/fpsyg.2022.897463

**Published:** 2022-06-27

**Authors:** Wenming Zhang

**Affiliations:** Department of Philosophy & Literature and History, Shenzhen Party School & Shenzhen Academy of Governance, Shenzhen, China

**Keywords:** cultural entertainment consumption, interest-related community, sports film, community discourse, empathy communication

## Abstract

The economic and cultural effects of sports films have attracted close attention from academia as well as the industry. In this paper, two sub-studies were conducted to explore the empathy mechanism performance of the interest-related community in sports films. In Study 1, the film *Lead* was applied as an example and used network text analysis to analyze the discourse characteristics and structure of its interest-related community to grasp the practice regularities. More specifically, the results in Study 1 show that the theme feature, brand building, technological innovation, and spirit communication are the key factors that affect an individual's empathy for sports films. In Study 2, I conducted a survey to explore the empathy mechanism of sports films in the new age to provide a theoretical reference for the high-quality development of films. The above-mentioned four main factors have positive impacts on empathy: affective empathy and cognitive empathy.

## Introduction

As the information application level of social life improves, the individuals conduct more convenient exchanges and communication, and an interconnected community structure has taken shape. Driven by the development of the Internet, the exchange of individuals' interests has broken through the regional shackles and presented the characteristics of the new era (Wang et al., 2020a, 2021; Xu et al., 2020; Li et al., 2021a; Deng et al., 2022). The development of information technology and big data availability enables individuals to make a more diverse evaluation of consumption (Wang et al., 2020b; Zhang S. et al., 2020; Lee and Peng, 2021; Li et al., 2021b; Yi et al., 2021a; Geng et al., 2022). Films are facing multiple influences and impacts from the characteristics of community discourses, and the development of the film industry is confronting new opportunities and challenges (Yi et al., 2020). Among various types of films, sports films are a special branch of Chinese films, closely related to sports and have a long history, such as *Queen of Sports, Women Basketball Player No. 5, Girls on the Ice, Female Divers*, and *Young Swallow* are world-renowned, and the cultural spirit contained in them has inspired generations of enthusiastic audiences. In the past National Day market competition, *Lead* engaged the whole population with sports IP, with a box office of over RMB 100 million and an excellent reputation. It was crowned as the pioneering work of sports-themed blockbusters in the new age and made the audiences burst into tears with the spirit. The discourse characteristics and practice mode of the interest-related community of *Lead* are worthy of in-depth exploration by the academic community.

Today, in the context of the rapid evolution of economic growth, individuals have a growing need for a better life, the demand for cultural and entertainment consumption has risen to a higher level, and interactive experience has become the core requirement of film watching in the new age. The individuals are no longer limited to visual and audio enjoyment, but in the pursuit of collision of psychology and emotion, and the empathy of local cuisines, song, film, and television has become the key factor for the success of destination management (Li and Katsumata, 2020; Yin et al., 2020; Cao et al., 2021; Yi et al., 2022). Specifically, a large number of films watching evaluation feedbacks show that the effective empathy communication system presented by the film contents not only ensures effective transmission of the film's popularity but also improves economic and social benefits (Griffin et al., 2022). The films are developing toward high quality and high standards recognized by the public. As a strong market competitor for the films screened during the National Day holiday in 2020, *Lead* touched countless Chinese audiences. As a leader in Chinese films that can stimulate the audience's psychological and emotional resonance, its empathy mechanism has presented a new film mode.

Accordingly, sports films are presenting new demands and challenges in the context of the new age, and the empathy mechanism that impresses the audience's psychology and emotion has become a key issue that requires in-depth attention (Barber, 2014; Rodesiler, 2014; Yi et al., 2021b). Given that, this paper took the film *Lead* as an example and conducted Study 1 to analyze the discourse characteristics and structure of its interest-related community to grasp the practice regularities and Study 2 to explore the empathy mechanism of sports films in the new age through a survey on film watching to provide a theoretical reference for the high-quality development of films of the kind in China.

## Study 1: Literature Review and Research Questions

### Films and Cultural Consumption

Since its inception, films have received widespread attention, and can be classified into three categories: feature films, expansion films, and documentaries (Connell, 2012; Du et al., 2020; Teng and Chen, 2020; Marques de Sousa et al., 2021). In the primary stage, the idea of educating people through sports resulted in a narrative form with a focus on recording, supplemented with empathy and experience (Baker, 1998). On this basis, scholars have carried out academic research on the theme of sports, successively made explorations on the screen culture related to issues of nation, country, and esthetics, and officially launched the integration of sports, short video, and films (Barber, 2014; Rodesiler, 2014; Ferreira et al., 2022). As an integral part of the film family, sports film has many fans, and its research has attracted extensive attention from experts in various fields of academia. Scholars began to focus on the design of narrative and video, and successively explored in detail from such perspectives as macro narrative, style analysis, and narrative analysis, and the concept of “sports IP” was nurtured and incubated, becoming a new wave of research on the film contents (Barber, 2014; Levental, 2021; Siyu, 2021). Scholars have started a new round of research boom in film from aspects of the film-induced tourism, cultural implication, and cultural identity (Du et al., 2020; Griffin et al., 2022).

### Interest-Related Community and Film and TV Communication

As an important path for cultural communication, the film is a key area rooted in cultural development. How to achieve scientific, efficient, and accurate film and television communication has always been a key issue in the field of communication in various periods (Kretz, 2019; Ju, 2020; Fang, 2021). With the rise of the Internet, people are accelerating to establish more interpersonal “circles,” forming a complex network of relationships. The concept of community has attracted extensive attention from scholars, and a consensus has been reached to explore the essential issues of film and television communication; the community interaction of the audiences of film and television communication ought to be clarified so that the inherent laws and characteristics can be defined (Riddle and Martins, 2022).

The film community belongs to the human relationship formed under the virtual boundary. It is a collection of human beings gathered due to the interest in watching films, implying a unified relationship between the space of discourse practice and the space of spiritual interaction (Nikolaeva et al., 2018; Ayikoru and Park, 2019; Kubrak, 2020). Therefore, most existing studies have carried out related explorations from the perspective of community discourse texts and spiritual expression. To better analyze the characteristics of films, previous studies have attempted to explain the content characteristics, narrative logic, and cultural connotation of films through the language symbols and situational symbols in the community discourses (Bo, 2018; Newell, 2018). Moreover, in a bid to further explore the humanistic spirit contained in the films, film research has increasingly focused on culture to construct the spirit of community through case analysis or example analysis of the times and regional characteristics (Addeo et al., 2019). As the dialectical thinking about entertainment issues in film studies increases, the interest-related community is becoming a focus in film and television communication research, and the discourse textual analysis and spiritual and cultural analysis relating to communities are attracting more attention.

### Empathy Mechanism in the Film Community

The characteristics of people-to-people interaction in the community, the characteristics of individual experience, and the resulting increase in benefits have become the key issues. Multiple needs emerge in the film community age and the audience's psychological and emotional resonance after watching a film needs to be deeply explored (Rodrigues and Loureiro, 2022). Psychological and emotional resonance is one of the topics in psychological research, and its inherent similar feelings among individuals and between individuals and things have been interpreted as the concept of empathy by academics (Mehrabian and Epstein, 1972; Cuff et al., 2016).

Empathy refers to a phenomenon in which the individuals project their spiritual feelings on objective things (Mehrabian and Epstein, 1972). In the communication after watching the film, empathy is obvious and representative. After the audience watches the film, more frequent evaluation and communication will inevitably occur in the context of the Internet, and the transmission of information will continue to ferment in the interest-related community of the film, as the role of interests continues to increase (Jeong et al., 2021). The spiritual world that is urgently needed to be described in the film provides a deeper experience for the film audiences and an imaginative space for those who have not watched the film (Quintal and Phau, 2015). In the age of the film community, individuals' diverse needs present empathic characteristics, and the empathy mechanism demonstrates the core role of the film community interaction.

Overall, with the accelerating pace of economic development, individuals are paying increasing attention to sports films, and the internal psychological and emotional changes in sports film consumption are becoming a new heated topic. Based on the focus and preference of film and television communication and the performance of diversified needs in the new age, this study took the film *Lead* as an example to conduct systematic research on the following three issues:

*Q1: The factors of interest-related community discourse and empathic features in sports films*.
*Q2: The practice rules of interest-related community discourse in sports films*.
*Q3: Analysis of the empathy mechanism of interest-related community in sports films*.

### Research Design

#### Research Methods

This study adopted qualitative and quantitative research methods to conduct combinatory analysis. The network text analysis method was applied to analyze the inherent semantics of the high-frequency words in the film *Lead*, and then the categorization analysis and encoding were carried out based on the Grounded Theory to construct the feature hierarchy of factors. Furthermore, the research integration method was applied for qualitative analysis to put forth the factors and dimensions of the discourse practice of the interest-related community of the film *Lead* and construct the empathy mechanism model of the interest-related community in line with the empathy theory. Finally, the structural equation model analysis method was applied to verify the assumptions and conclusions proposed in this study. The in-depth macro and microanalyses were conducted to realize the scientific research on the practice and empathy mechanism of the interest-related community discourse of sports films.

#### Technical Path

**Figure 1:**
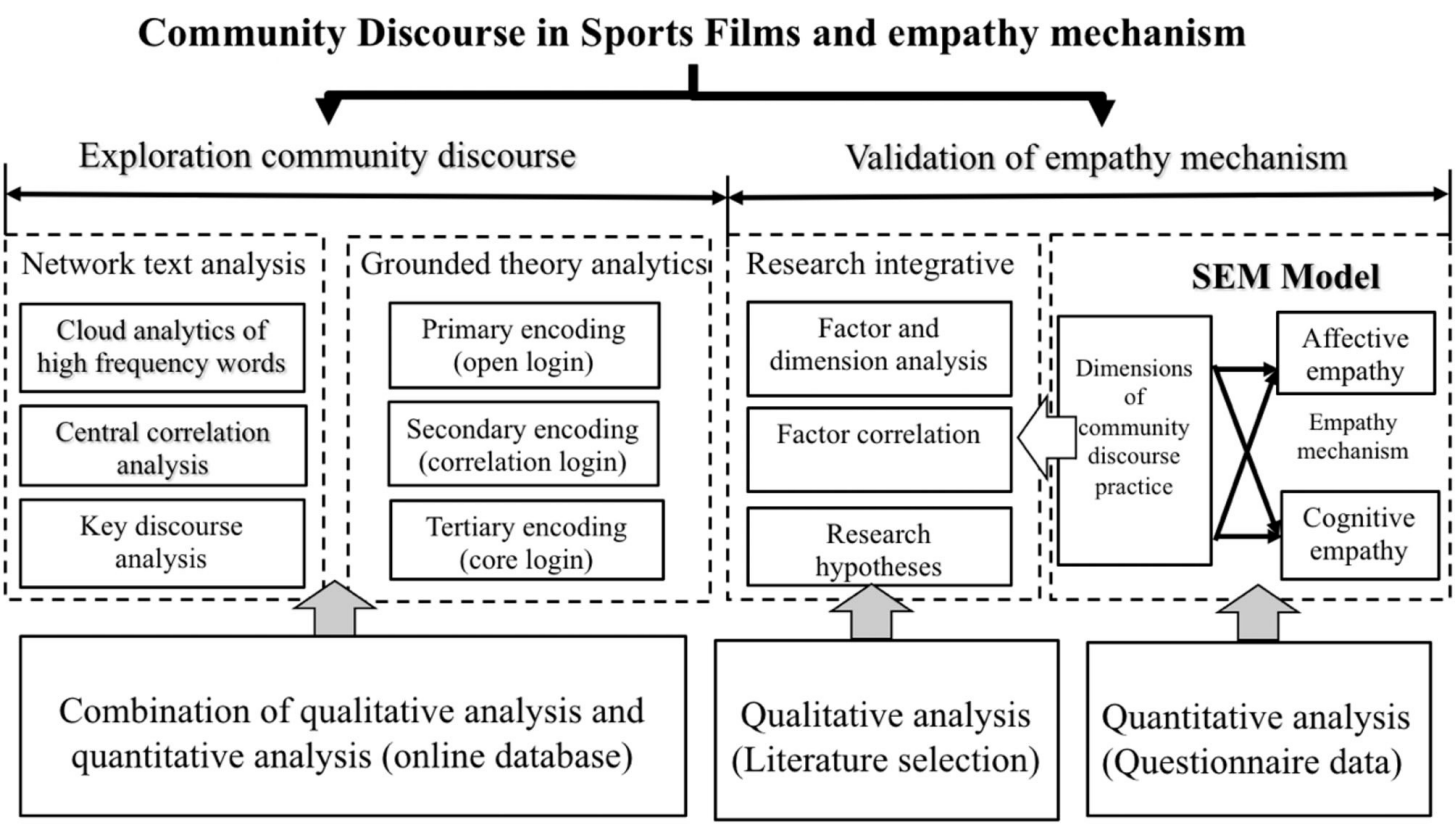
Technical path map.

This study conducted regularity exploration and validation research, including common factor analysis, mechanism construction, and empirical analysis. The research technical path was shown in Figure 1. First, the ROST-CM6 software was used to search the entire online text data related to the film *Lead* with “lead” and “film” as the keywords, and excavate the discourse characteristics of the interest-related community based on the composition of coding elements of the Grounded Theory; second, based on the analysis of the characteristics of community discourses, the research integration method was applied to clarify the internal empathy logic of the interest-related community of films and put forward relevant research hypotheses and the theoretical structural framework of the empathy mechanism; third, the SmartPls software was adopted to verify the characteristics of the interest-related community discourses and empathy mechanism presentation of sports films; finally, the empirical analysis results and logical exploration conclusions were taken account of to draw the research conclusions and implications.

#### Feature Encoding of Interest-Related Community Discourse in Sports Films

The ROST-CM6 software was applied to conduct an in-depth analysis of 1,635,910 pieces of online information related to the keywords from September 25, 2020, to October 8, 2020. According to the linear change characteristics of the number of discourse samples, the film data on the first day of its debut reached its peak, with a total of 291,825 pieces of information.

#### Cloud Analytics of High-Frequency Words

Based on the discourse characteristics of the film community, keywords irrelevant to this study were picked out and excluded, and synonymous keywords were merged, for instance, “film,” “champion,” and “championship,” “women's volleyball,” “Chinese women's volleyball,” and “volleyball,” “film ticket” and “box office,” “watching films” and “cinema,” “Gong Li,” “actor,” “Bai Lang,” “Wu Gang,” and “Huang Bo,” “Chen Kexin” and “director,” “shooting” and “photography,” which were merged into “film,” “champion,” “women's volleyball,” “box office,” “actor,” “director,” and “shooting” as the discourse presentation. Based on this, the top 50 keywords were finally selected for the high-frequency word analysis (as shown in Table 1).

**Table 1:** High-frequency words of network texts of the film *Lead*.

**Ranking**	**High frequency words**	**Count**	**Word class**
1	Women's volleyball	1,996,441	Noun
2	Film	1,989,448	Noun
3	Champion	1,861,627	Verb
4	Box office	1,158,261	Noun
5	Actor	1,150,306	Noun
6	China	813,901	Noun
7	Screening	704,187	Verb
8	Director	668,140	Noun
9	Spirit	531,543	Noun
10	Shooting	419,097	Verb
11	Story	338,550	Noun
12	Audience	321,828	Noun
13	Honor	311,807	Noun
14	Lang Ping	284,503	Noun
15	Athlete	249,979	Noun
16	Authenticity	247,001	Noun
17	Sense of mission	242,117	Noun
18	Character	237,736	Noun
19	Reshape	217,728	Verb
20	Display	217,250	Verb
21	Team	216,392	Noun
22	Detail	211,199	Noun
23	Visual	195,525	Noun
24	Appear on the stage	193,452	Verb
26	Base	190,843	Noun
27	Moment	182,961	Noun
28	Weibo lottery	182,456	Noun
29	Zhangzhou	182,105	Noun
30	Screenwriter	180,031	Noun
31	Arduous	177,684	Adjective
32	Clipping	176,405	Verb
33	Adapting	175,446	Verb
34	Material	175,004	Noun
35	Uncle	170,649	Noun
36	PETE	170,604	Noun
37	Filtering	169,737	Verb
38	Prototype	167,041	Noun
39	Art	166,914	Noun
40	Match	164,159	Noun
41	Select	163,999	Verb
42	Struggle	162,438	Verb
43	Revere	161,583	Verb
44	One by one	158,953	Pronoun
45	Endless	158,535	Adjective
46	Do magic	157,926	Verb
47	Win	133,083	Verb
48	Jingdong	123,274	Noun
49	Goal	112,303	Noun
50	Extract	106,226	Verb

The keywords repeatedly mentioned by audiences in the process of discussion and evaluation on online platforms are the prominent key features of the interest-related community discourse practice in sports films. Given that, the current study attempted to observe the characteristics of community discourse practice through the extracted keywords. Specifically, the keywords were mostly nouns and verbs. Among the high-frequency words, the top three “women's volleyball,” “film,” and “champion” were the theme words of the film, further highlighting that the discourse characteristics of the interest-related community paid frequent attention to the theme. Among the keywords, “box office” and “audience” ranked the 4th and 12th, respectively, which specifically reflected that the society was concerned with the economic effect of films, and as is true to all kinds of films, the box office is the core indicator to intuitively indicate the quality of the film. Additionally, “actor,” “director,” “audience,” “character,” “team,” and “screenwriter” stood at the 5th and 30th among the keywords, demonstrating that the film participants are widely concerned. Moreover, film technical actions such as “shooting,” “clipping,” “adapting,” and “filtering” also occupied a substantial proportion among the top 50 high-frequency words in the whole online texts, and technical application is becoming the key point of evaluation of film and television works in the new age. In addition, nouns such as “spirit” and “sense of mission,” verbs such as “struggle” and “revere,” and adjectives such as “arduous” and “endless” jointly demonstrated the core feature of sports films, namely, spirit communication.

Overall, all the high-frequency words in the whole online texts of the film *Lead* not only expounded the common needs of the film works and the brand building generated by the participants but also showed the new requirements of films on technology and innovation in the new age, and additionally, they also indicated the characteristics of sports films presented with sports elements. Based on this, the factors of the interest-related community discourse practice of the film *Lead* can be divided into four dimensions: theme feature, brand building, technological innovation, and spirit communication.

#### Central Correlation and Key Discourses Analysis

**Figure 2:**
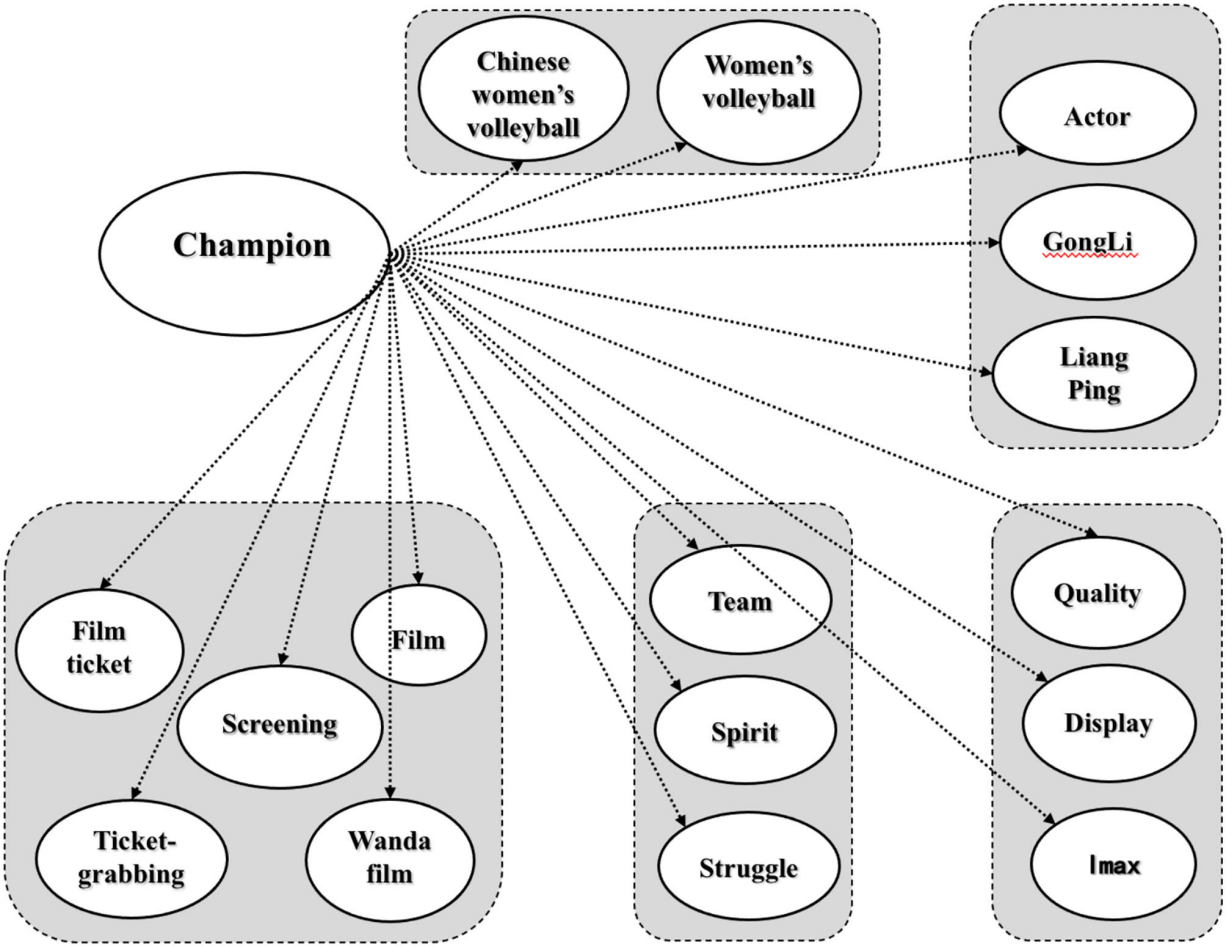
Discourse correlation in the film *Lead*.

To further analyze and clarify the continuity characteristics reflected in things, explore the association rules that cannot be directly explained in semantic interpretation, and explore the deep structural characteristics of the interest-related community, this study extracted the relationship network centered on “champion” to reflect the relationship between factors, dimensions, and the target center based on intuitive analysis of high-frequency words, and then to deeply understand the characteristics of the interest-related community discourse practice of sports films. The semantic network of the ROST-CM6 software was applied (as shown in Figure 2) and found that the film *Lead* formed a typical five-dimension performance, specifically, the economic and social effect indicator represented by “screening,” “film,” “film ticket,” “ticket-grabbing,” and “Wanda film,” which was closest to “champion” and showed the most concerned topic in the interest-related community discourse; the theme feature factor represented by “Chinese women's volleyball” and “women's volleyball,” which was the second closest to “champion” and was a vital factor to interpret the interest-related community discourse practice in sports films. The cultural spirit factor represented by “team,” “spirit,” and “struggle” demonstrated the unique spirit communication factor of sports films and was the key factor in the discourse practice of interest-related community in sports films; the keywords such as “actor,” “Gong Li,” and “Lang Ping” were still closely related to “champion,” indicating that the actors were still a main influencing factor, which reflected the brand building as independent characters in the film; the viewing experience derived from technological innovation represented by “quality,” “display,” and “imax” had gradually come to the fore and became another important factor in the interest-related community discourse practice in sports films. Based on this, this study further interpreted the dimensional characteristics reflected in the cloud analytics of high-frequency words through the discourse correlation of the film *Lead* and deepened the understanding of the correlation between the four dimensions including theme feature, brand building, technological innovation, and spirit communication and the interest-related community discourse practice in sports films. Moreover, this study highlighted that the effect was still the main indicator of the evaluation of sports films, and the mechanism structure of the discourse practice of the interest-related community in sports films was clearly defined.

#### Categorization Analysis

In the online text data, whether sensitive, neutral, or non-sensitive words, all reflected the practice characteristics of the interest-related community discourse in sports films. As the data were collected from diversified media sources such as Weibo, Client (Workstation), WeChat, websites, forums, news, videos, newspapers, and governmental portals, they can evaluate the demand characteristics of films in multiple aspects. Because of this, the data composed of 1,635,910 pieces of information were too large; thus, the current study only analyzed the top 50 high-frequency words of the online texts obtained to provide a theoretical basis for defining the future development of sports films.

The Grounded Theory was adopted to conceptualize and encode all the keywords based on cloud analytics of the high-frequency words and central correlation and key discourse analysis to extract the internal core categories. Through the three-level coding process “open login → association login → core login”, this study extracted 20 secondary factors, including sports theme, film theme, spirit theme, IP building, image building, content enriching, star effect, marketing strategy, creation technology, shooting technology, production technology, projection technology, consumption link, passion, inspiration, unity, self-transcendence, dream fulfillment, economic effect, and social effect, and five generic dimensions including theme feature, brand building, technological innovation, spirit communication, and comprehensive effect (see Table 2).

**Table 2:** Encoding process of community discourse in the sports film *Lead*.

**Primary encoding** **(open login)**	**Secondary encoding** **(correlation login)**	**Tertiary encoding** **(core login)**
Women's volleyball	Sports theme	Theme feature
Film, screening	Film theme	
Champion	Spirit theme	
Screenwriter, material, match	IP shaping	Brand building
Lang Ping, athlete, prototype, reshaping	Image building	
Story, authenticity, character	Content enriching	
Actor, director	Star effect	
Base, Weibo lottery platform, Jingdong	Marketing strategy	
Reshaping, adapting, selecting	Creation technology	Technological innovation
Shooting	Shooting technology	
Clipping, detail, visual, music, filtering, art	Production technology	
Displaying	Screening technology	
One by one, endless	Consumption link	
Spirit, uncle (disseminator), Pete (disseminator)	Passion	Spirit communication
Struggle, revere	Inspiration	
China, team	Solidarity	
Sense of mission, appear, on the stage, arduous	Self-transcendence	
Honor, moment, win	Dream fulfillment	
Box office	Economic effect	Comprehensive effect
Audience, Zhangzhou (publicity effect), do magic	Social effect	

## Study 2: Empathy Mechanism in Sports Films and Research Hypotheses

### Sports Film and Empathy Theory

Involving economic operation laws such as production, marketing, and consumer demand, films are cultural and entertainment products for improving economic benefits (Craig et al., 2005). The sports elements contain complex social and cultural connotations and are an important symbol of social development, and thus sports films bear the cultural dissemination of sports and are a key element to enhance social cohesion (Simoni, 2017). Nowadays, the cultural efficacy of sports films is highly valued by academia which began to focus on the characteristics of cultural value other than the economic value of sports films. In the process of making sports films in China, a logical connection is being formed between the film consumption market and the creation of cultural value (Koh et al., 2010).

**Figure 3:**
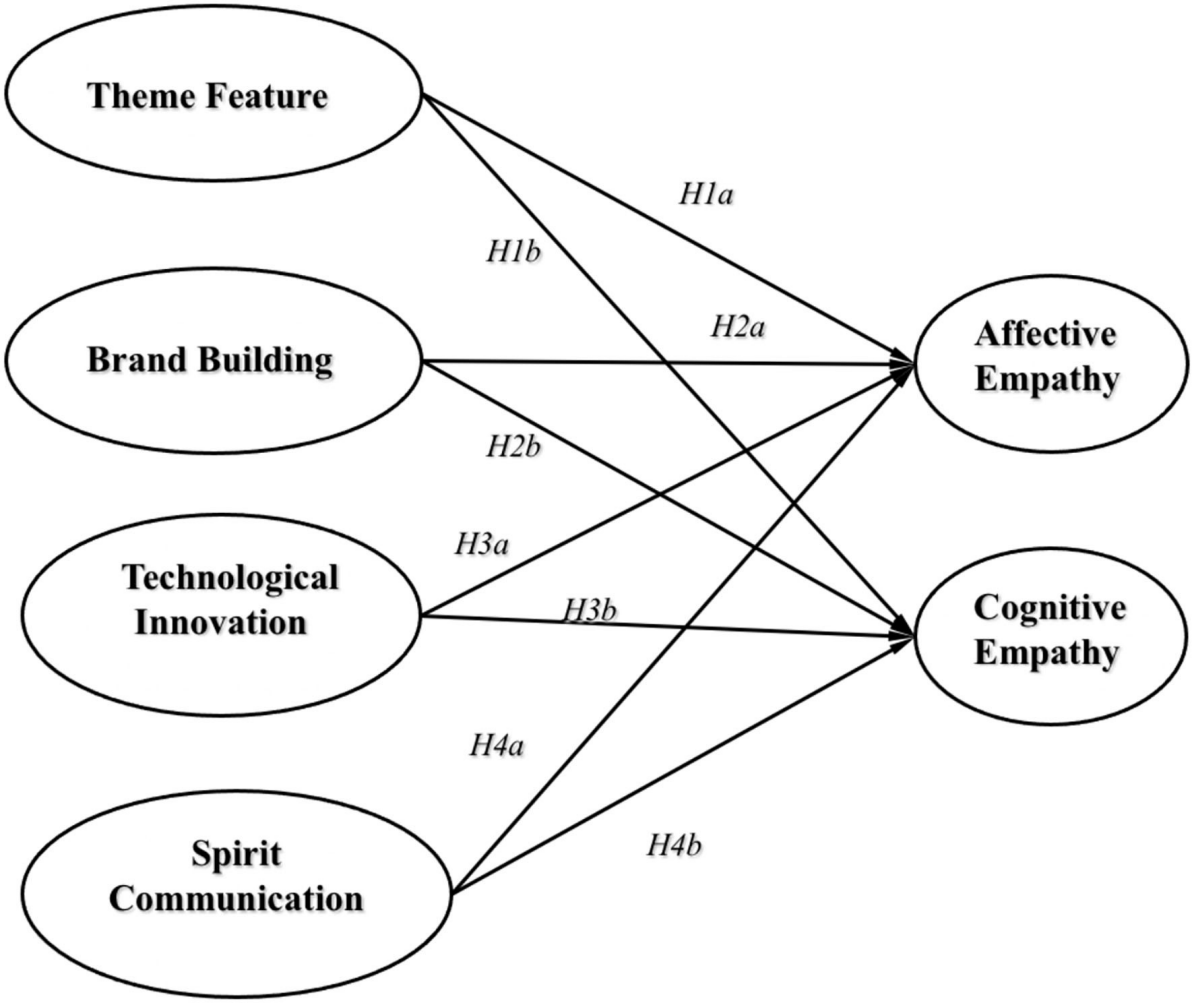
Empathy mechanism model.

In the field of psychology, empathy is an important theory for explaining psychological and emotional problems (Blair, 2005; Yi et al., 2021b). It originated from the sensory performance of interpreting the empathy of art products to describe the emotional communication between people and objects or individual interaction (Boler, 1997). The high-quality development of sports films should be based on the psychology of the audience and focus on their internal needs to produce high-quality products that adapt to market competition. Based on the process performance of cognitive empathy and affective empathy, and taking phenomena and characteristics as a reference, this study applied the guiding logic of the audience's psychological and emotional characteristics to the future development direction of sports films, and the empathy mechanism model of this study is shown in Figure 3.

### Empathy and Community Discourse in Sports Films

The interest-related community discourse in the context of sports films is a side manifestation of the film-watching behavior, and it is also a direct presentation of the behavior after the movie consumption. Therefore, the regular exploration of the interest-related community discourse practice can better explain the psychological and emotional changes of the film audience. Specifically, sports films are affecting and changing people's emotional contagion degree, regularities of emotional changes, and emotion recognition effect, as well as people's cognition of ideology, expectation, emotion, and perspective in many ways.

In sports films, the typical sports theme makes sports enthusiasts more susceptible to empathy, show positive emotional changes, and better understand and recognize their emotional performance (Rodrigues and Loureiro, 2022). Sports films are still of traditional modes and have the inherent emotional effects of films, which can manipulate the audience's emotions through the screen language and enable the audience to gain sensory experience in the virtual scene (Daniels, 2005). Besides, the screen language also involves a large amount of knowledge, which can promote the audience to increase their knowledge and ability in unknown areas, supplement and enhance the existing ideological cognition, expectation cognition, emotional cognition, and perspective cognition; thus, the film themes will comprehensively enhance the audience's multi-faceted understanding of scientific angles (Umanath et al., 2012). Furthermore, with the rapid increase in the level of demand for films, apart from film theme and sports theme, spirit theme has begun to take shape in sports films. This new theme originated from the producers' domestic demand for cultural communication and emotional expression, and it is becoming a content element of films to influence the audience's emotions (Srinivasan and Martinez, 2018). Therefore, the thematic features in sports films are a necessary condition for affective empathy and cognitive empathy, which can effectively change the audience's emotional contagion, affective changes, and emotional recognition performance, and improve the audience's ideological, expectation, emotional, and perspective cognition. Given that, the following hypotheses are proposed:

*H1a: The theme features of sports films have a positive effect on affective empathy*.
*H1b: The theme features of sports films have a positive effect on cognitive empathy*.

As a kind of cultural and entertainment consumer product, sports films also have brand effects. The highest level of the brand effect is the resonance of consumers with the core value of the product in the form of association, consensus, and symbiotic logic, which are the main evaluation indexes for empathy (Rodrigues and Loureiro, 2022). In addition, character-shaping is often accompanied in sports films, and the characters with representative characteristics are often the essence and cornerstone of films. Previous studies have shown that image building in sports films often directly reflects the internalized culture and spirit in films, which is the intuitive expression of the highest level of films (Zhang and Zhai, 2018). Besides, the idea that “the content is paramount” is a core topic throughout the development history of the film industry. Audiences often have film viewing behaviors out of the preference of a film star, making the audience's emotional contagion and recognition faster, affective changes more vivid, and the cognitive performance more rational (Pu et al., 2010). Moreover, apart from the influence of the film itself, the marketing before the film screening and the marketing methods during the screening will make the audience close to the film and empathy with the film in an earlier, better, and more accurate way to produce an emotional resonance effect (Westbury and Neumann, 2008). Therefore, brand building in sports films is a necessary condition for affective empathy and cognitive empathy, which can effectively change the audiences' emotional contagion, affective changes, and emotional recognition performance, and improve their ideological, expectation, emotional, and perspective cognition. Accordingly, the following hypotheses are put forth:

*H2a: The branding building in sports films has a positive effect on affective empathy*.
*H2b: The branding building in sports films has a positive effect on cognitive empathy*.

With the introduction of the concept of film and television industrialization, the application of film technological innovation has become a vital part of film development in the new age and has begun to replace cultural creativity as the mainstream direction. From a psychological and emotional perspective, technological innovation in film and television includes the innovative application of creative technology, the process of which is often related to human needs and is the process of the creators' imagination and shaping of the audience's film and television needs (Quitzow, 2015). The improvement of creative technology can effectively stimulate emotions and steer their positive changes while deepening and clarifying people's core cognition (Edele et al., 2013).

Film and television technological innovation also include the innovative implementation of production technology, which is an integral link between creation and shooting during production, transformable and adjustable based on original materials, and it is an effective regulator to deepen or reduce the degree of the audience's emotional and psychological changes (Samoilov and Goldfried, 2000). The consumption link is the extension of film and television technological innovation, which can make people's entertainment consumption more diversified and allow them to have a more in-depth experience, richer and clearer emotional infection and recognition, more sensitive emotional changes, and clear and rational cognition of thoughts, expectation, sentiments, and viewpoints (Cheng et al., 2021). Technological innovation in sports films is a necessary condition for affective empathy and cognitive empathy, which can effectively change the audience's emotional contagion, affection, and emotional recognition performance, and improve the ideological, expectation, emotional, and perspective cognition. Accordingly, the following hypotheses are proposed:

*H3a: Technological innovation in sports films has a positive effect on affective empathy*.
*H3b: Technological innovation in sports films has a positive effect on cognitive empathy*.

Self-transcendence and achievement of dreams are the product of the combination of sports films and social life, which is a higher expression of people's emotions, and a key factor for the gradual maturity of cognition and a lasting effect on normal life (Green et al., 2008). Spirit communication in sports films is a necessary condition for affective empathy and cognitive empathy. It can effectively change the audiences' emotional contagion, sentiment, and emotional recognition performance, and improve their ideological, expectational, emotional, and perspective cognition (Penza and Cassano, 2004). Accordingly, the following hypotheses are put forward:

*H4a: Spirit communication in sports films has a positive effect on affective empathy*.
*H4b: Spirit communication in sports films has a positive effect on cognitive empathy*.

### Empathy Mechanism of Interest-Related Community in Sports Films

#### Model Construction

**Figure 4:**
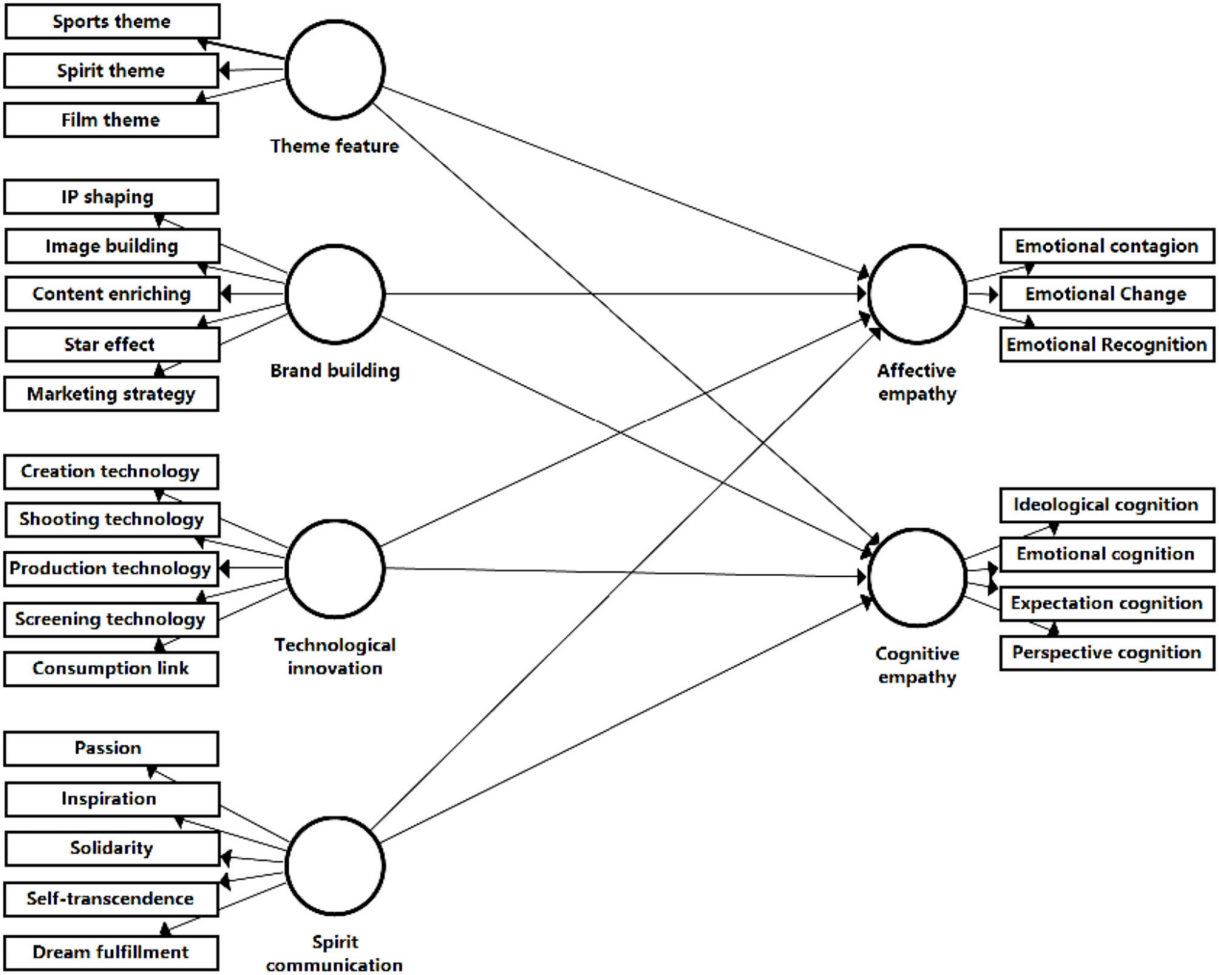
Conceptual model of the structural equations.

Based on the element encoding representation of interest-related community discourse practice in sports films and the hypotheses of the empathy mechanism of interest-related community in sports films, this study formed the interpretation items guided by the feature encoding, the element association oriented by the hypotheses and then constructed a complete structural equation model framework, namely four independent variables, including theme feature (independent variable) jointly interpreted by spirit theme, film theme, and sports theme. Brand building (independent variable) was jointly interpreted by IP modeling, image building, content enriching, star effect, and marketing strategy. Technological innovation (independent variable) was jointly interpreted by creation technology, shooting technology, production technology, projection technology, and consumption link. Spirit communication (independent variable) was jointly interpreted by passion, inspiration, solidarity, self-transcendence, and dream fulfillment. In addition, two dependent variables including affective empathy jointly expounded by emotional contagion, affective change, and emotional recognition, and cognitive empathy jointly expounded by ideology, expectation, emotional, and perspective cognition. Accordingly, the empirical model of the empathy mechanism of the interest-related community in sports films is constructed (as shown in Figure 4).

#### Data Analysis and Exploration Analysis

Based on the specific current situation in the film *Lead* and in line with the characteristics of the interest-related community discourse practice of sports films, the current study adopted the method of previous studies (Wang and Yi, 2020; Zhang L. et al., 2020) of the 5-point Likert scale to design the questionnaire and conducted a survey of various audiences who have watched the film *Lead*. A total of 351 questionnaires were distributed and 342 (male = 146, 42.7%) valid questionnaires were received, with a recovery rate of 97.4%, the detailed information was shown in Table 3.

**Table 3:** Descriptive statistics of respondents' characteristics (*n* = 342).

**Demographics**	**Option**	**Count**	**Percentage**
Gender	Male	146	42.7%
	Female	196	57.3%
Age	Below 20 years old	125	36.5%
	21–30 years old	142	41.5%
	31–40 years old	44	12.9%
	Above 41 years old	31	9.1%
Occupation	Technical	67	19.6%
	Research	68	19.9%
	Art	55	16.1%
	Management	86	25.1%
	Sociality	39	11.4%
	Routine	27	7.9%
Education	Junior high school and below	24	7.0%
	High school/technical secondary school	74	21.6%
	Junior college	37	10.8%
	Undergraduate	163	47.7%
	Postgraduate and above	44	12.9%
Marital Status	Single	141	41.2%
	In love	116	33.9%
	Married	63	18.4%
	Divorced or widowed	22	6.4%
Monthly consumption (RMB)	<3,000	182	53.2%
	3,000–5,000	92	26.9%
	5,001–10,000	46	13.5%
	More than 10,000	22	6.4%

Based on the test, this study adopted the principal component analysis method to explore the regularities of the questionnaire data. The extraction eigenvalue was set >1. Under the method of Kaiser normalization maximal rotation of variance, it converged after 14 iterations to form a 4-component matrix. The variance ratio of each component was 20.632, 18.574, 18.400, and 12.981%, respectively, and the cumulative value was 70.587%, which met the research requirements. This study selected four dimensions, including theme feature, brand building, technological innovation, and spirit communication. All the dimensions showed significant component characteristics, and the independent variables and the interpretation items were established.

This study carried out the reliability and validity test. The statistical analysis of reliability was as follows: the Alpha values of theme feature, brand building, technological innovation, spirit communication, affective empathy, and cognitive empathy were 0.844, 0.885, 0.890, 0.893, 0.839, and 0.871, respectively. The Alpha value of each variable was >0.7 and indicating that each latent variable had strong reliability. And the composite reliability value was >0.9, further proving that the model had high reliability; and the AVE of each latent variable were >0.5, all in the tolerance interval. The results of validity analysis r was as follows: KMO value was 0.987 and the value of Bartlett's test of sphericity was 7,178.361, indicating that the data were highly scientific and effective and can be used to explore the regularities. The above analysis suggested that the overall model fit was satisfactory, the internal latent relationships had a significant explanatory performance, the estimated effect was acceptable, and the reliability indices were consistent with the construct validity. The detailed information is shown in Table 4.

**Table 4:** Validity and reliability of measures.

**Variables**	**Cronbach's Alpha**	**rho_A**	**C.R**.	**AVE**
Theme feature	0.844	0.844	0.906	0.763
Brand building	0.885	0.886	0.916	0.686
Affective empathy	0.839	0.839	0.903	0.757
Cognitive empathy	0.871	0.872	0.912	0.722
Technological innovation	0.890	0.890	0.919	0.694
Spirit communication	0.893	0.895	0.921	0.701

#### Validation Analysis of Structural Equation Model

Based on exploratory factor analysis, this study adopted the SmartPls software to conduct a validation analysis of the structural equation model. In addition, in the sample (*n* = 342), the four-factor model showed overall goodness of fit of the structural equation model (see Table 5), SRMR was 0.038 (<0.08), d_ULS was 0.464 (<0.95), d_G was 0.437 (<0.95), and NFI was 0.892 (>0.8); thus, all indicators showed a high level of goodness of fit, passing the test of goodness of fit. Therefore, the overall goodness of fit of the model was high, and the research conclusions were rational and effective.

**Table 5:** Goodness-of-fit measures.

	**Evaluation standard**	**Model parameter**	**Goodness- of-fit**
SRMR	0.038	<0.08	Good fit
d_ULS	0.464	<0.95	Good fit
d_G	0.437	<0.95	Good fit
χ^2^	798.318		
NFI	0.892	>0.8	Good fit

**Figure 5:**
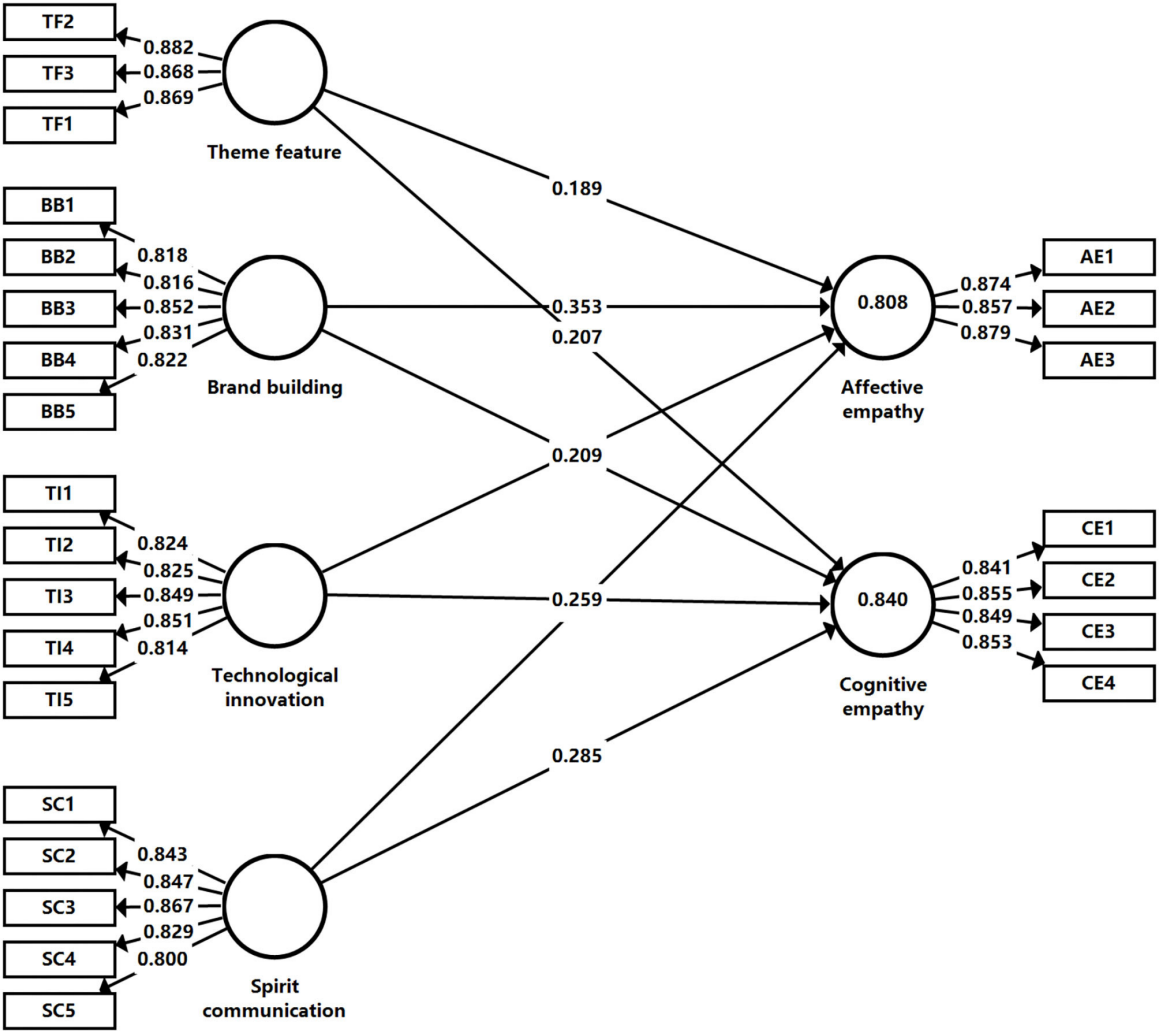
Empathy mechanism of interest-related community.

The Bootstrapping method was used to calculate the T statistic for each path coefficient to test the significance level of each path coefficient estimate (two-tailed test), as shown in Table 6. The T statistic of the structural equation model of this study in the Bootstrapping test showed that all path coefficients were with high T statistic. Specifically, in terms of the estimate value, theme feature → affective empathy was 0.189, theme feature → cognitive empathy was 0.207, brand building → affective empathy was 0.353, brand building → cognitive empathy was 0.209, technological innovation → affective empathy was 0.238, technological innovation → cognitive empathy was 0.259, spirit communication → affective empathy was 0.161, and spirit communication → cognitive empathy was 0.285. In addition, the *P*-value of each path was <0.05, indicating that each path coefficient passed the test of the corresponding significance level, and the model had a stable structure. In terms of item explanatory, values were shown in Figure 5, the estimate coefficients were all >0.5, passing the validation test. The coefficient indexes in Figure 5 demonstrated that the hypotheses proposed in this study were all established.

**Table 6:** Bootstrapping: results of pathway coefficients.

	**Original sample (O)**	**Sample mean (M)**	**Standardized deviation (STDEV)**	***T*-Statistic (|O/STDEV|)**	***P*-value**
Theme feature -> Affective empathy	0.189	0.184	0.061	3.073	0.002
Theme feature -> Cognitive empathy	0.207	0.211	0.053	3.895	0.000
Brand building -> Affective empathy	0.353	0.352	0.066	5.357	0.000
Brand building -> Cognitive empathy	0.209	0.209	0.057	3.700	0.000
Technological innovation-> Affective empathy	0.238	0.243	0.066	3.616	0.000
Technological innovation-> Cognitive empathy	0.259	0.255	0.056	4.624	0.000
Spirit communication -> Affective empathy	0.161	0.162	0.064	2.502	0.013
Spirit communication -> Cognitive empathy	0.285	0.285	0.059	4.872	0.000

## Research Conclusions and Implications

### Research Conclusions

This study conducted two sub-studies to explain the practice characteristics of the interest-related community discourse in sports films and explore the regularities of the audience's empathy mechanism through the case study of film *Lead*. To comprehensively explore and validate the interest-related community discourse in sports films, multimethodology was conducted, including network text analysis, grounded theoretical analysis, integrative analysis, and empirical analysis.

The results of empirical analysis show that H1, H2, H3, and H4 were supported. Thus, the research hypotheses are all established, and the theme feature, technological innovation, and spirit communication have a more significant effect on cognitive empathy, and brand building has a more significant effect on affective empathy. Therefore, taking the comprehensive improvement of theme features, brand building, technological innovation, and spirit communication as the overall strategy is the development path for sports films in line with the needs of the times. Specifically, this study puts forward four suggestions for the future development of sports films. First, the finding indicates that the development direction of sports films should comprehensively consider the thematic elements of sports, films, and spirits to make the film themes originate from life and outline a thematic picture that can enhance individuals' cognition, beat their hearts, and make them empathetic. Second, the findings indicate that the effect of brand building on affective empathy is much stronger than that on cognitive empathy, while content enrichment still dominates the development of sports films. Therefore, the development of sports films in the future should still adhere to the strategy that “the content is paramount” and create film brands that can touch people's moods to further improve the audiences' viewing experience and achieve sustainable high-quality development. Third, in terms of path coefficient, coefficients and factor loadings indicate that while the technological innovation of films is constantly being emphasized, the technological innovation of various aspects of sports films is still at an equal level, and the whole-process technological innovation should be considered in future research development.

### Research Implications

The study provided several research implications. First, the interest-related community discourse in sports films generally has five dimensions of performance, namely theme feature, brand building, technological innovation, spirit communication, and comprehensive effects. Second, this study extracted and formed the interest-related community discourse characteristics of sports films, that is, through the characteristics of independent variables including theme feature, brand building, technological innovation, and spirit communication, relevant interpretation items were analyzed and selected. Specifically, the theme feature includes sports theme, film theme, and spirit theme; brand building includes IP building, image building, content enriching, star effect, and marketing strategy; technological innovation includes creation technology, shooting technology, production technology, projection technology, and consumption link; spirit communication includes passion, inspiration, solidarity, self-transcendence, and dream fulfillment. Third, this study explored and analyzed the important characteristic elements of empathy in sports films, namely emotional contagion, affective change and emotional recognition, ideological cognition, expectation cognition, and sentiment cognition under the dual-process dimension of affective empathy and cognitive empathy.

Accordingly, this study enriched the literature in sports films by constructing the empathy mechanism of interest-related community, forming a systematic structure from the four factors of theme feature, brand building, technological innovation, and spirit communication to the two factors of affective empathy and cognitive empathy. The results show that the spirit communication of sports films can significantly improve individuals' ideological cognition, expectation cognition, emotional cognition, and perspective cognition, which is the key to improving individuals' perception of social life. Therefore, to improve the contents of sports films more effectively, we should further strengthen the understanding of consumers' cognitive performance feedback, closely follow the “solidarity” element, and create high-quality sports films with a profound and positive connotation.

## Data Availability Statement

The original contributions presented in the study are included in the article/supplementary material, further inquiries can be directed to the corresponding author/s.

## Ethics Statement

The studies involving human participants were reviewed and approved by Shenzhen Party School & Shenzhen Academy of Governance. Written informed consent from the [patients/participants OR patients/participants legal guardian/next of kin] was not required to participate in this study in accordance with the national legislation and the institutional requirements.

## Author Contributions

The author confirms being the sole contributor of this work and has approved it for publication.

## Conflict of Interest

The author declares that the research was conducted in the absence of any commercial or financial relationships that could be construed as a potential conflict of interest.

## Publisher's Note

All claims expressed in this article are solely those of the authors and do not necessarily represent those of their affiliated organizations, or those of the publisher, the editors and the reviewers. Any product that may be evaluated in this article, or claim that may be made by its manufacturer, is not guaranteed or endorsed by the publisher.
